# Are cells from a snowman realistic? Cryopreserved tissues as a source for single-cell RNA-sequencing experiments

**DOI:** 10.1186/s13059-017-1192-4

**Published:** 2017-03-24

**Authors:** Felipe A. Vieira Braga, Sarah A. Teichmann, Michael J. T. Stubbington

**Affiliations:** 1 Wellcome Trust Sanger Institute, Wellcome Genome Campus, Hinxton, Cambridge, CB10 1SA UK; 2 European Molecular Biology Laboratory, European Bioinformatics Institute (EMBL-EBI), Wellcome Genome Campus, Hinxton, Cambridge, CB10 1SD UK; 3 Cavendish Laboratory, Cambridge University, JJ Thomson Avenue, Cambridge, CB3 0HE UK

## Abstract

A recently published study in *Genome Biology* shows that cells isolated from cryopreserved tissues are a reliable source of genetic material for single-cell RNA-sequencing experiments.

Please see related Method article: http://genomebiology.biomedcentral.com/articles/10.1186/s13059-017-1171-9

## Single-cell RNA-sequencing: an ongoing revolution

Single-cell RNA-sequencing (scRNA-seq) protocols have developed at a rapid pace in recent years. The first scRNA-seq protocol to be published generated libraries from up to 16 cells over six days [1]. Now, thousands of cells can be isolated and prepared ready for sequencing in one or two days using droplet microfluidics technologies [2] or conventional flow cytometry coupled with automated liquid handlers [3]. Technical improvements have led not only to an increase in the number of cells analyzed simultaneously, but also to continuous reduction in cost per cell. This progress has increased throughput and has contributed to democratizing scRNA-seq technologies. These new scRNA-seq methods open the possibility of obtaining a better understanding of diverse biological systems. In this issue of *Genome Biology*, Heyn and colleagues study the possibility of using cryopreserved tissues in scRNA-seq experiments [4].

New biological insights provided by scRNA-seq include the identification of populations of mouse retinal cells [2], a map of the developing mammalian heart and programs involved in congenital heart disease [5], and new CD4+ T cells responsible for steroid production [6]. The diversity in biological systems illustrates the revolutionary power of current scRNA-seq protocols.

In addition to technical improvements in scRNA-seq protocols, the development of computational tools to analyze the large datasets generated are vital to the generation of novel biological insights. scRNA-seq data analysis has its own challenges when compared to bulk RNA-seq analysis, and specific tools designed for quality control, data exploration, clustering and visualization [2] are essential to generate useful biological insights. Other recent developments include the reconstruction of T-cell receptor sequences [7], which permits the analysis of T-cell clonality and transcriptional identity in parallel, and the unraveling of developmental processes by analyzing dynamic changes in gene expression and ordering the cells in pseudo-time [8].

## From rare samples to single cells

Despite the diversity of the computational scRNA-seq methods that are now available and the variety of biological systems studied by them, most studies rely on the use of fresh cells and tissues. Modern-day biological research is highly collaborative and often involves several experiments taking place across multiple locations, separated by large distances; the necessity of using fresh cells and tissues is a limiting factor for such studies at the single-cell level. This is a limitation especially when studying infectious diseases such as Ebola and malaria, as the patients who donate samples are often located thousands of kilometers away from the scientists analyzing the biological materials. Furthermore, complex experimental design can also lead to a time gap between tissue collection and the actual experiment, as is the case, for example, when antigen-specific T cells need to be isolated using specific tetramers, a process that demands prior human leukocyte antigen (HLA) genotyping of the tissue donor. Thus, fresh samples are not always available and alternative methods to preserve tissue in a way that is compatible with scRNA-seq technologies are needed. Previous work has shown that cryopreservation of brain tissue is compatible with sequencing of RNA isolated from single nuclei [9] but, until now, there has not been evidence of successful scRNA-seq using cryopreserved whole cells.

## Extending analysis to frozen cells

Cryopreservation of tissues and cells in dimethyl sulfoxide (DMSO) is a method widely used for the conservation of biological samples. The paper by Heyn and colleagues [4] presents a detailed study on the feasibility of using cryopreserved tissues and cells as a source of material for scRNA-seq. The authors compare single-cell transcriptomic data obtained using cell lines that were freshly sequenced or sequenced after freezing and thawing. Despite differences in cell viability, both samples had comparable numbers of sequencing reads and detected genes. Dimensionality reduction via principal component analysis (PCA) and t-distributed stochastic neighbor embedding representations (t-SNE) show similarity between fresh and cryopreserved samples. These results are consistent for both 3′ MARS-seq and full-length Smartseq2 scRNA-seq methods, suggesting that cryopreserved cells might be a valuable source of material for different scRNA-seq experiments.

One of the greatest possibilities created by scRNA-seq technologies is the unbiased analysis of cell populations within complex and heterogeneous tissues. Heyn and colleagues [4] extended their analysis to human peripheral blood mononuclear cells (PBMCs), mouse colon tissue and ovarian carcinoma. scRNA-seq analysis of cryopreserved PBMCs was capable of identifying all of the major immune subsets (B cells, monocytes, T cells and NK cells). Analysis of murine colon identified transit amplifying cells, secretory enteroendocrine cells and differentiated enterocytes in both fresh and cryopreserved samples in similar proportions.

However, within the T-cell subpopulation structure in blood, the proportions of memory and cytotoxic cells vary between fresh and cryopreserved samples. A difference in subpopulation proportions was also observed in tumor samples. This might be due to different populations being affected differently by freezing or to technical biases introduced by different times of sampling. Considering that the authors showed that cell lines do not change their transcriptome upon freezing, such small biases are most probably due to differences in the capacity of different cells to survive cryopreservation methods. This suggests that, independent of the technique used to analyze such samples, small biases in population proportions might occur in cryopreserved samples.

Thus, one should always carefully consider the experimental design and maintain consistency across samples for a defined tissue source, as direct comparison of fresh versus cryopreserved samples might lead to different conclusions. As with so many things, optimization of protocols for individual tissues will be important.

## The road ahead

The possibility of using cryopreserved tissues in scRNA-seq experiments will certainly expand the range of possible experimental designs and biological questions that can be addressed. However, further developments in this area are still needed. Current DMSO-based cryopreservation methods are compatible with work in research settings but will be harder to implement within the workflow of diagnostic and tissue bank services, as most samples are directly fixed using formalin or another fixative method. Some initial attempts to perform scRNA-seq using fixed single-cell suspensions as the source material [10] have generated promising results. Hence, systematic evaluation of the suitability of fixed tissues or different methods of cryopreservation for scRNA-seq experiments are the next frontier to be explored.

## Abbreviations

DMSO: Dimethyl sulfoxide
PBMC: Peripheral blood mononuclear cell
scRNA-seq: Single-cell RNA-sequencing
